# Is the cardio-immune microenvironment a convergent hub of cardiovascular toxicity across novel anticancer therapies?

**DOI:** 10.3389/fphar.2026.1980237

**Published:** 2026-10-29

**Authors:** Youzhuo Quan, Chunlan Lang, Peiliang Xie, Yun Zhang

**Affiliations:** 1 Department of Radiology, West China Hospital, Sichuan University, Chengdu, China; 2 Department of Cardiology, Union Hospital, Tongji Medical College, Huazhong University of Science and Technology, Wuhan, China; 3 Outpatient Department, West China Hospital, Sichuan University, Chengdu, China; 4 West China School of Medicine, Sichuan University, Chengdu, China

**Keywords:** antibody–drug conjugates, cancer therapy–related cardiovascular toxicity, cardio-immune microenvironment, cardio-oncology, CAR-T-cell therapy, endothelial dysfunction, immune checkpoint inhibitors

## Abstract

Novel anticancer therapies have transformed oncology but have also expanded the spectrum of cancer therapy–related cardiovascular toxicity, including myocarditis, ventricular dysfunction, hypertension, vascular inflammation, thrombosis, arrhythmias, fibrosis, and heart failure. In cardio-oncology, the cardio-immune microenvironment provides a useful framework for integrating cardiovascular toxicity across immune checkpoint inhibitors, CAR-T cell therapy and other T-cell–redirecting therapies, HER2-directed therapies, VEGF/VEGFR inhibitors, antibody–drug conjugates, radiotherapy-containing regimens, and combination treatments. This Review discusses the cardio- immune microenvironment as a tissue-level network in which immune cells, cardiomyocytes, endothelial cells, vascular cells, fibroblasts, soluble mediators, and extracellular-matrix cues interact during injury and repair. Rather than proposing a universal immune origin for all cardiotoxic effects, we distinguish three routes by which novel anticancer treatments may enter this network: immune initiation, systemic inflammatory translation, and primary cardiomyocyte, endothelial, vascular, or payload-related injury followed by immune amplification and remodeling. We define mechanistically meaningful convergence as engagement of a shared downstream cellular–molecular response module by therapies with distinct proximal mechanisms, while distinguishing such convergence from nonspecific injury–repair responses. We organize the cardio-immune framework around five mechanistic domains: immune tolerance breakdown, cytokine-driven systemic inflammation, sterile danger sensing, myeloid and stromal reprogramming, and endothelial–immune coupling. We further discuss how these mechanisms may inform therapy-specific risk stratification, biomarker interpretation, imaging surveillance, mechanism-guided intervention, and multi-omics approaches in precision cardio-oncology. This context-dependent framework may help clarify why distinct anticancer therapies can produce overlapping cardiovascular phenotypes while preserving therapy-specific mechanisms and clinical management priorities.

## 1 Introduction

Modern oncology has moved beyond a single cytotoxic paradigm and now includes therapies that mobilize antitumor immunity, engineer T cells, inhibit defined oncogenic pathways, or deliver cytotoxic payloads through tumor-directed antibodies (Johnson et al., 2016; June and Sadelain, 2018; Min and Lee, 2022; Liu et al., 2024). This therapeutic expansion has reshaped cancer care, but it has also broadened the cardiovascular safety questions faced by oncologists and cardiologists. Cancer therapy-related cardiovascular toxicity is now recognized as a heterogeneous clinical spectrum that includes cardiomyopathy or heart failure, myocarditis, vascular toxicity, hypertension, arrhythmias, QTc prolongation, and thromboembolic complications (Lyon et al., 2022; Herrmann et al., 2022).

Early models of cardiotoxicity focused mainly on direct injury to cardiomyocytes or vascular cells. Trastuzumab can dysregulate HER2 signaling and impair basal autophagy in human primary cardiomyocytes (Mohan et al., 2016). Inhibitors of the vascular endothelial growth factor pathway are associated with hypertension and heart failure (Touyz and Herrmann, 2018). These examples show that some cardiovascular toxicities arise from therapy-specific disruption of cardiomyocyte survival pathways, endothelial homeostasis, or vascular regulation (Mohan et al., 2016; Touyz and Herrmann, 2018). However, a cardiomyocyte-centered or vessel-centered view is not sufficient to explain the full biological complexity of cardiovascular injury after cancer therapy. Cardiovascular toxicity therefore needs to be considered at the tissue level, where cardiomyocyte, vascular, immune, and stromal responses may interact during injury and repair.

The role of immune dysregulation is most evident in immune checkpoint inhibitor-associated myocarditis. Immune checkpoint inhibitors (ICIs) have improved clinical outcomes in numerous malignancies, but severe immune-related adverse events can occur, particularly with combination immunotherapy. In patients who developed fatal myocarditis after combined CTLA-4 and PD-1 blockade, myocardial inflammatory infiltrates included T lymphocytes and macrophages. The same report identified shared or expanded T-cell clones in myocardium, skeletal muscle, and tumor tissue, supporting the possibility of antigen-driven immune injury involving striated muscle (Johnson et al., 2016). Experimental evidence that PD-L1 expressed on cardiac endothelial cells controls immune-mediated cardiac injury further supports the importance of immune-checkpoint pathways in cardiac immune tolerance (Grabie et al., 2007).

CAR-T-cell therapy provides a second, mechanistically distinct example of immune-associated cardiovascular injury. Cytokine release syndrome (CRS) is a major toxicity of T-cell-based immunotherapies and involves inflammatory cytokine networks that can activate immune cells and non-immune cells (Shimabukuro-Vornhagen et al., 2018). In adults treated with CAR-T cells, cardiac injury and cardiovascular events were reported after therapy, and a graded relationship was observed among CRS, troponin elevation, and cardiovascular events (Alvi et al., 2019). These findings connect systemic immune activation with cardiovascular injury, but they also highlight an unresolved question: how does a circulating inflammatory response become translated into tissue-specific cardiac dysfunction?

Together, these observations suggest that the cardio-immune microenvironment, defined here as the tissue-level interface linking immune processes with both myocardial and vascular/endothelial compartments, may serve as a conceptual bridge between therapy-specific cardiovascular insults and shared downstream phenotypes. However, the presence of overlapping inflammatory, vascular, or remodeling responses across therapies does not by itself establish mechanistic convergence, because similar responses may also arise as generic consequences of tissue injury and repair. A central question is therefore when shared downstream responses represent biologically meaningful convergence and when they merely reflect nonspecific injury–repair programs. This framework does not imply that all cardiovascular toxicities induced by novel anticancer therapies are primarily immune-mediated; some therapies enter this network through primary cardiomyocyte stress, endothelial dysfunction, or vascular injury, as illustrated by HER2-directed therapy and VEGF-pathway inhibition (Mohan et al., 2016; Touyz and Herrmann, 2018). Rather, we examine how immune processes may initiate cardiovascular injury in selected contexts, amplify primary cardiomyocyte or vascular damage in others, and regulate inflammatory resolution, fibrosis, and remodeling after tissue injury (Swirski and Nahrendorf, 2018; Adamo et al., 2020). By distinguishing immune initiation from immune amplification and repair/remodeling regulation, this review aims to determine when therapy-specific insults engage shared downstream cardio-immune response modules, when such overlap represents generic tissue repair, and how mechanistically meaningful convergence can be empirically distinguished from nonspecific injury responses.

## 2 The cardio-immune microenvironment as a tissue-level platform for injury and remodeling

The cardio-immune microenvironment can be understood as a dynamic tissue niche in which resident and recruited immune cells interact with cardiomyocytes, endothelial cells, fibroblasts, vascular cells, and extracellular-matrix components. This concept is supported by anatomical and single-cell studies showing that the adult heart is a heterogeneous multicellular organ with substantial cellular diversity across human cardiac regions (Pinto et al., 2016; Litvinukova et al., 2020). Within this local environment, immune cells are not merely transient inflammatory effectors. Resident cardiac macrophages contribute to tissue surveillance and homeostatic regulation, whereas recruited monocytes and macrophages participate in inflammation, tissue repair, regeneration, and fibrosis after cardiac injury (Lafuse et al., 2020; Epelman et al., 2014).

Cardiac injury activates tightly connected immune, stromal, vascular, and cardiomyocyte responses. Cardiomyocyte death and extracellular-matrix degradation can release danger-associated signals that activate innate immune pathways, induce cytokine and chemokine production, and promote leukocyte recruitment (Frangogiannis, 2014a). This early inflammatory response facilitates clearance of dead cells and supports tissue repair, but excessive or unresolved inflammation may amplify injury and contribute to adverse remodeling (Frangogiannis, 2014a; Forte et al., 2018). Fibroblasts and myofibroblasts are central stromal effectors in this process. In response to inflammatory, mechanical, vascular, and cardiomyocyte-derived signals, activated fibroblasts and myofibroblasts produce extracellular-matrix proteins and drive fibrotic remodeling (Frangogiannis, 2021).

This tissue-level framework is particularly relevant to cardiovascular toxicity induced by novel anticancer therapies. Although different therapies may initiate injury through distinct proximal mechanisms, including immune activation, cytokine release, endothelial stress, oxidative injury, or direct cardiomyocyte dysfunction (Herrmann, 2020), the observation of overlapping downstream responses does not by itself establish mechanistic convergence. Shared responses such as damage-associated molecular pattern release, pattern-recognition receptor activation, sterile inflammatory signaling, or endothelial and vascular activation may represent mechanistically relevant intermediate processes in some treatment contexts but generic injury–repair biology in others (Klee et al., 2017). This distinction requires a more explicit operational definition of cardio-immune convergence.

We therefore define cardio-immune convergence as the engagement of a shared cellular–molecular response module within cardiovascular tissues by two or more anticancer therapy classes with mechanistically distinct proximal routes of injury. Such convergence should not be inferred solely from shared clinical phenotypes or from the presence of cytokine release, leukocyte recruitment, fibroblast activation, or extracellular-matrix remodeling, which may represent generic injury–repair responses. We consider a shared response a candidate convergence node when it is engaged across mechanistically distinct therapies, is positioned between the proximal insult and downstream cardiovascular phenotype, and is temporally associated with injury amplification, persistence, resolution, or remodeling. This definition generates a testable prediction: if a shared node represents mechanistically meaningful rather than nonspecific convergence, its activation should precede or accompany toxicity progression, and its perturbation should modify the cardiovascular injury trajectory across at least two mechanistically distinct treatment contexts. These criteria provide a framework for evaluating the candidate pathways discussed below.

## 3 Cardio-immune convergence across novel anticancer therapies

Diverse anticancer therapies enter the cardio-immune microenvironment through distinct proximal mechanisms. ICIs primarily disrupt immune tolerance, CAR-T-cell therapy and other T-cell–redirecting therapies link systemic immune activation to cardiovascular stress, whereas targeted therapies and antibody–drug conjugates (ADCs) more often initiate cardiomyocyte, endothelial, vascular, or payload-related injury (Palaskas et al., 2024; Force et al., 2007; Mahalingaiah et al., 2019). These distinct routes provide the mechanistic contrast required to distinguish true cross-therapy convergence from parallel manifestations of injury (Figure 1). Accordingly, this section maps the predominant proximal route associated with each treatment class (Table 1), whereas shared downstream response modules are evaluated in Section 4.

**FIGURE 1:**
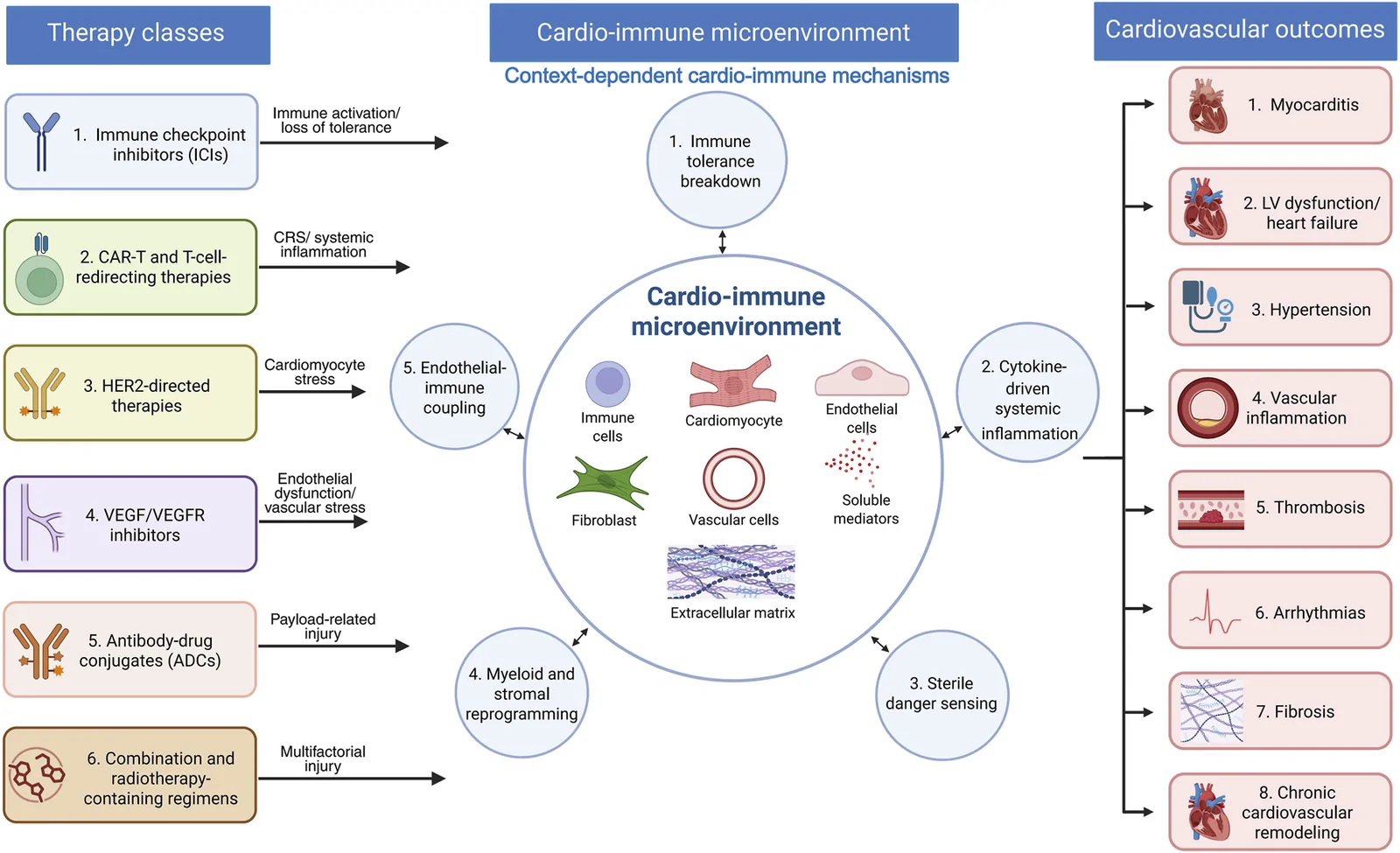
Therapy-specific injury mechanisms and context-dependent cardio-immune responses across novel anticancer therapies. Novel anticancer therapies enter the cardio-immune microenvironment through therapy-specific proximal routes, including loss of immune tolerance, systemic inflammation, cardiomyocyte stress, endothelial/vascular stress, payload-related injury, and multifactorial injury. ICIs exemplify immune-initiated cardiovascular injury, whereas HER2-directed therapies, VEGF/VEGFR inhibitors, and ADCs primarily cause cardiovascular tissue injury with potential secondary immune amplification; CAR-T-cell and T-cell–redirecting therapies predominantly involve systemic inflammatory translation. Within this network, immune and cardiovascular cells interact through immune tolerance breakdown, cytokine-driven inflammation, sterile danger sensing, myeloid/stromal reprogramming, and endothelial–immune coupling. These processes may contribute to myocarditis, LV dysfunction/heart failure, hypertension, vascular inflammation, thrombosis, arrhythmias, fibrosis, and chronic cardiovascular remodeling. Abbreviations: ADCs, antibody–drug conjugates; CAR-T, chimeric antigen receptor T-cell; CRS, cytokine release syndrome; ICIs, immune checkpoint inhibitors; LV, left ventricular; VEGF, vascular endothelial growth factor; VEGFR, vascular endothelial growth factor receptor.

**TABLE 1:** Therapy-specific routes into the cardio-immune microenvironment across novel anticancer treatments.

Treatment class	Primary route of entry	Predominant cardio-immune process	Evidence base	Framework interpretation	References
Immune checkpoint inhibitors	Loss of immune tolerance after CTLA-4 or PD-1/PD-L1 blockade	T-cell–mediated injury and multicellular inflammatory niche	Human myocardial tissue and immune profiling; clinical cohorts; experimental models	Best-supported immune-initiated cardiovascular toxicity	Mahmood et al. (2018), Wei et al. (2021), Axelrod et al. (2022), Blum et al. (2024)
CAR-T-cell and T-cell–redirecting therapies	Systemic immune activation and CRS	Cytokine-driven inflammation, myeloid amplification, and endothelial activation	Clinical cohorts and biomarker studies; mechanistic experimental models	Systemic inflammatory translation with endothelial and hemodynamic involvement	Alvi et al. (2019), Lefebvre et al. (2020), Giavridis et al. (2018), Norelli et al. (2018), Maleki et al. (2024), Hay et al. (2017)
HER2-directed therapies	Disruption of HER2/ErbB2-dependent cardiomyocyte homeostasis	Primary cardiomyocyte stress with secondary inflammatory/remodeling responses	Experimental cardiomyocyte and animal evidence; clinical cardiovascular phenotype; limited direct human tissue immune evidence	Secondary immune/remodeling modifier rather than established immune initiation	Mohan et al. (2016), Grazette et al. (2004), Crone et al. (2002)
VEGF/VEGFR and angiogenesis inhibitors	Endothelial dysfunction and impaired vascular homeostasis	Endothelial–immune coupling and vascular inflammatory activation	Clinical vascular phenotype and mechanistic vascular studies; limited direct myocardial tissue evidence	Predominantly vascular injury with potential secondary immune amplification	Touyz and Herrmann (2018), Lankhorst et al. (2014), Herrmann et al. (2016), Terwoord et al. (2022)
Antibody–drug conjugates	Target-, linker-, and payload-related tissue injury	Potential sterile danger sensing and secondary immune amplification	Construct biology and preclinical mechanistic evidence; clinical safety data; limited direct cardiovascular tissue evidence	Construct-specific primary injury with potential downstream cardio-immune amplification	Mahalingaiah et al. (2019), Donaghy (2016)
Combination therapies and radiotherapy-containing regimens	Concurrent immune, myocardial, vascular, or radiation-related injury	Multifactorial immune, endothelial, stromal, and fibrotic responses	Clinical trials/meta-analyses and toxicity studies; radiobiological and experimental mechanistic evidence; limited cross-therapy causal evidence	Natural experiments for convergence; synergy is not established by increased toxicity alone	Liu et al. (2022), Inno et al. (2024), Koutroumpakis et al. (2022), Philipp et al. (2020), Mitchell et al. (2021)

Representative references indicate selected studies or reviews supporting the proposed route of entry and dominant cardiovascular toxicity pattern for each treatment class, rather than an exhaustive citation list. Abbreviations: CAR-T, chimeric antigen receptor T-cell; CRS, cytokine release syndrome; CTLA-4, cytotoxic T-lymphocyte-associated protein 4; HER2, human epidermal growth factor receptor 2; ICI, immune checkpoint inhibitor; PD-1, programmed cell death protein 1; PD-L1, programmed death-ligand 1; VEGF, vascular endothelial growth factor; VEGFR, vascular endothelial growth factor receptor.

### 3.1 Immune checkpoint inhibitors: breaking immune tolerance in the heart

ICIs restore antitumor immunity by blocking inhibitory immune pathways such as CTLA-4 and PD-1/PD-L1, which normally restrain T-cell activation (Ribas and Wolchok, 2018). ICI-associated myocarditis is uncommon but high-risk, often occurs early after treatment initiation, and is associated with major adverse cardiac events in reported cohorts (Mahmood et al., 2018). Pharmacovigilance studies have also linked ICIs to a broader spectrum of cardiovascular immune-related toxicities, including myocarditis, pericardial disease, arrhythmias, and vasculitis (Salem et al., 2018).

Within the cardio-immune framework, ICIs represent a prototypical immune-centered route because checkpoint blockade can disturb immune tolerance itself and permit activated T cells and inflammatory pathways to enter cardiovascular tissues (Ribas and Wolchok, 2018). Thus, immune-tolerance breakdown is best considered a therapy-specific initiating mechanism rather than, by itself, evidence of cross-therapy convergence; whether downstream immune, myeloid, stromal, or vascular responses subsequently converge with those induced by other therapies is addressed in Section 4.

### 3.2 CAR-T and cellular therapies: systemic inflammation meets cardiac immunity

CAR-T therapy is an adoptive cellular therapy in which patient-derived or donor-derived T cells are engineered to recognize tumor-associated antigens through synthetic antigen receptors (June and Sadelain, 2018). CRS is one of the major acute toxicities of CAR-T-cell therapy and reflects excessive immune activation after therapeutic T-cell engagement (Neelapu et al., 2018). Cardiovascular complications frequently occur in the setting of CRS, and higher CRS severity has been associated with troponin elevation and major cardiovascular events in observational cohorts (Alvi et al., 2019; Lefebvre et al., 2020).

In contrast to the direct disturbance of immune tolerance seen with ICIs, CAR-T-cell therapy primarily enters the cardio-immune microenvironment through systemic inflammatory translation: therapeutic T-cell activation triggers cytokine and myeloid responses that can impose hemodynamic, endothelial, and myocardial stress (Shimabukuro-Vornhagen et al., 2018; Neelapu et al., 2018). Thus, any convergence between ICIs and T-cell–redirecting therapies would occur downstream of distinct proximal immune triggers rather than through an identical initiating mechanism.

### 3.3 Targeted therapies and antibody–drug conjugates: primary tissue injury with secondary immune amplification

Targeted therapies and ADCs should be distinguished from immune-based therapies when discussing cardio-immune convergence. For many targeted therapies, cardiovascular toxicity begins with disruption of signaling pathways required for cardiomyocyte or vascular homeostasis rather than with primary immune activation (Force et al., 2007). ADC-related toxicity requires a further distinction because organ injury may reflect target biology, linker instability, premature payload release, or target-independent uptake of the intact conjugate (Mahalingaiah et al., 2019; Donaghy, 2016). Thus, in this context, the cardio-immune microenvironment is best viewed as an amplifier and regulator of tissue injury rather than as the primary trigger.

HER2-targeted therapy illustrates a cardiomyocyte-centered form of primary injury. Cardiomyocyte-specific loss of ErbB2 causes dilated cardiomyopathy in experimental models, and antibody-mediated ErbB2 inhibition can impair mitochondrial function in cardiomyocytes, supporting a direct role for HER2/ErbB signaling in myocardial homeostasis (Grazette et al., 2004; Crone et al., 2002). Accordingly, secondary inflammatory or stromal responses following HER2-directed injury should not automatically be interpreted as evidence of cardio-immune convergence, particularly when prominent inflammatory infiltration is not demonstrated.

VEGF/VEGFR inhibition represents a vascular-centered route. VEGF blockade can promote hypertension through disruption of nitric oxide signaling and endothelin-1 activation (Lankhorst et al., 2014), while VEGF signaling more broadly supports endothelial homeostasis and vascular regulation (Touyz and Herrmann, 2018; Herrmann et al., 2016).

Other TKIs similarly illustrate how inhibition of pathways required for cardiac or vascular homeostasis can generate primary tissue stress: imatinib has been linked experimentally to mitochondrial and endoplasmic-reticulum abnormalities and cardiomyocyte death, whereas sunitinib has been associated clinically with hypertension and heart failure (Kerkelä et al., 2006; Chu et al., 2007). For ADCs, payload class, linker properties, and target-independent uptake further shape the dominant toxicity phenotype (Mahalingaiah et al., 2019; Donaghy, 2016). Across these therapies, shared downstream immune or stromal responses constitute meaningful convergence only if they can be distinguished from expected secondary responses to primary tissue injury.

### 3.4 Combination therapies: a natural test of the convergence hypothesis

Combination regimens provide a clinically relevant setting in which distinct proximal insults coexist, but increased toxicity during combination therapy should not itself be considered evidence of mechanistic convergence or synergy. Dual immune checkpoint blockade has been associated with a higher frequency of severe treatment-related adverse events than single-agent checkpoint inhibition (Sznol et al., 2017). PD-1/PD-L1 inhibitors combined with chemotherapy increase all-grade and grade 3–5 cardiotoxicity compared with chemotherapy alone (Liu et al., 2022), consistent with coexisting tissue injury and immune activation but not proving interaction at a shared causal node.

Similarly, ICIs combined with angiogenesis inhibitors increase severe hypertension without significantly increasing acute vascular events (Inno et al., 2024), suggesting amplification of selected vascular phenotypes rather than uniform synergistic cardiotoxicity.

Radiotherapy-containing combinations add local endothelial, pro-thrombotic, inflammatory, and fibrotic injury to systemic immune or vascular stress, but direct evidence for shared causal downstream nodes remains limited (Koutroumpak et al., 2022).

Combination therapy should therefore be viewed as a natural experiment for, rather than proof of, cardio-immune convergence. Demonstrating convergence requires evidence that distinct proximal insults engage a shared intermediate response module and, ideally, that perturbing this module alters the cardiovascular trajectory. Section 4 evaluates the extent to which the proposed immune, myeloid, danger-sensing, stromal, and endothelial pathways satisfy these criteria across treatment classes.

## 4 Shared and therapy-specific cardio-immune mechanisms driving cardiovascular toxicity

Therefore, we propose that the cardio-immune microenvironment should not be regarded as a universal initiating mechanism for all forms of cancer therapy-related cardiovascular toxicity. Rather, it functions as a tissue-level interface through which therapy-specific insults may be amplified, resolved, or converted into chronic cardiovascular remodeling. In the following sections, we organize cardio-immune convergence into five mechanistic domains: immune tolerance breakdown, cytokine-driven systemic inflammation, sterile danger sensing, myeloid and stromal reprogramming, and endothelial–immune coupling with chronic remodeling. These mechanisms do not contribute equally across treatment classes and should not all be interpreted as established cross-therapy convergence nodes; rather, they span therapy-specific initiating mechanisms and candidate downstream response modules supported by different levels of human, clinical, and preclinical evidence.

### 4.1 Immune tolerance breakdown and antigen-directed cardiac inflammation

Immune tolerance breakdown is the most clearly established cardio-immune mechanism in immune checkpoint inhibitor-associated myocarditis. CTLA-4 and PD-1/PD-L1 regulate antitumor immunity at distinct stages: CTLA-4 mainly modulates early T-cell activation, whereas PD-1 primarily limits effector T-cell activity in peripheral tissues (Pardoll, 2012). Recent cross-species transcriptomic evidence further suggests that PD-1/PD-L1 signaling is not merely a tumor-immune pathway, but also an endogenous regulatory axis within inflamed myocardial tissue (Yousif et al., 2026).

This mechanism is particularly relevant to the heart because immune checkpoint signaling appears to protect cardiovascular tissues from excessive T-cell-mediated injury. In a genetic mouse model, combined impairment of Ctla4 and Pdcd1 produced fatal myocarditis with immune-cell infiltration and features resembling ICI-associated myocarditis, supporting a functional interaction between these checkpoint pathways in cardiac immune homeostasis (Wei et al., 2021). These findings position ICI-associated myocarditis as a prototypical example in which anticancer immune activation directly enters the cardio-immune microenvironment by removing inhibitory constraints on autoreactive or tissue-damaging T cells.

Antigen-directed cardiac inflammation provides a more specific explanation for how tolerance loss can become myocardial injury. Impaired thymic tolerance to α-myosin has been shown to permit cardiac-directed autoimmunity, suggesting that some heart-specific antigens may escape complete central tolerance (Lv et al., 2011). In models of ICI-associated myocarditis, clonal effector CD8^+^ T cells recognizing α-myosin were identified as dominant pathogenic effectors, and α-myosin-expanded T cells from patients with ICI-associated myocarditis shared T-cell receptor clonotypes with diseased striated muscle tissues (Axelrod et al., 2022). A separate anti-PD-1 mouse model also demonstrated activation and expansion of cardiac myosin-specific autoimmune T cells, indicating that pre-existing antigen-experienced cardiac autoreactive T cells may become pathogenic after checkpoint blockade (Won et al., 2022).

However, ICI-associated myocarditis should not be reduced to a single model of shared tumor–heart antigen recognition. Single-cell and spatial immune profiling of human checkpoint myocarditis demonstrated enrichment and co-localization of cytotoxic T cells, conventional dendritic cells, inflammatory macrophages, and inflammatory fibroblasts within affected cardiac tissue (Blum et al., 2024). This indicates that tolerance breakdown is not only a T-cell-intrinsic event, but also a multicellular tissue process shaped by antigen presentation, myeloid activation, stromal inflammation, and local cardiac immune niches.

Within the broader cardio-immune convergence framework, immune tolerance breakdown should therefore be considered a dominant initiating mechanism for ICI-associated myocarditis rather than a universal mechanism across all anticancer therapies. Its importance lies in showing how antitumor immune activation can cross the boundary between tumor surveillance and cardiovascular tissue injury. Accordingly, immune tolerance breakdown is best regarded as a well-supported, ICI-dominant initiating mechanism rather than as a cross-therapy convergence node; potential convergence with other therapies is more likely to occur at downstream myeloid, stromal, or endothelial response modules.

### 4.2 Cytokine-driven systemic inflammation and cardiac–vascular translation

Cytokine-driven systemic inflammation is a major mechanism linking T-cell–redirecting therapies to cardiovascular injury. CAR-T-cell therapy and bispecific T-cell engager therapy can be complicated by CRS, a systemic inflammatory toxicity that may coincide with or trigger cardiovascular events in susceptible patients (Morris et al., 2022; Marar et al., 2023). CRS is the major acute toxicity of these therapies and is characterized by fever, hypotension, capillary leak, hypoxia, and multi-organ dysfunction in severe cases (Brudno and Kochenderfer, 2016).

This systemic inflammatory response can affect the cardiovascular system through several interconnected mechanisms. Elevated cytokines, including IL-6, IFN-γ, and TNF-α, can promote vasodilation, vascular permeability, and myocardial stress (Brudno and Kochenderfer, 2016; Ganatra et al., 2022). Preclinical studies further suggest that recipient myeloid cells, particularly monocytes and macrophages, are important sources of IL-1 and IL-6 during CAR-T-associated CRS (Giavridis et al., 2018; Norelli et al., 2018). These observations shift the mechanism from a purely T-cell-autonomous process toward a multicellular inflammatory circuit involving therapeutic T cells, tumor cells, myeloid cells, and cardiovascular tissues.

Clinical evidence supports the association between CRS and cardiovascular events after CAR-T therapy. An updated systematic review and meta-analysis reported cardiovascular events after CAR-T therapy, including arrhythmias, heart failure, cardiomyopathy, acute coronary syndrome, and cardiac arrest, and found that patients with cardiovascular events had a higher incidence of grade ≥ 2 CRS (Maleki et al., 2024). This relationship does not prove direct cytokine-mediated myocardial toxicity in every case, but it supports the concept that systemic inflammation and hemodynamic stress are major routes by which CAR-T therapy is translated into cardiovascular injury. Importantly, the resulting cardiovascular phenotype is likely multifactorial, reflecting interactions among systemic inflammation, hypotension, hypoxia, arrhythmias, endothelial dysfunction, and pre-existing cardiovascular vulnerability rather than direct cytokine-mediated myocardial injury alone.

Endothelial activation provides an important bridge between circulating inflammation and tissue-level cardiovascular dysfunction. In patients receiving CD19 CAR-T cells, severe CRS was associated with hemodynamic instability, capillary leak, consumptive coagulopathy, and increased biomarkers of endothelial activation, including angiopoietin-2 and von Willebrand factor (Hay et al., 2017). Through this pathway, systemic cytokine release may be converted into vascular permeability, microvascular dysfunction, thrombo-inflammatory stress, and secondary myocardial injury.

Bispecific T-cell engagers extend this mechanism beyond cellular therapy. These agents redirect endogenous T cells toward tumor cells and can produce CRS and cardiovascular adverse events, although current data remain less mature than for CAR-T therapy (Marar et al., 2023; Sayed et al., 2024). A recent pharmacovigilance study identified cardiovascular adverse events among reports involving bispecific T-cell engagers, and a large real-world cohort study found that cardiovascular event risk during T-cell engager therapy was higher in patients with pre-existing coronary disease or grade ≥2 CRS and/or immune effector cell-associated neurotoxicity syndrome (ICANS) (Sayed et al., 2024; Itzhaki Ben Zadok et al., 2026).

Therefore, cytokine-driven systemic inflammation should be viewed as a cardiac–vascular translation mechanism rather than simply a systemic adverse reaction. In this model, therapeutic T-cell activation initiates cytokine release, myeloid cells amplify inflammatory signaling, endothelial cells convert circulating inflammation into vascular dysfunction, and the myocardium experiences secondary stress through hypotension, tachyarrhythmia, impaired perfusion, and inflammatory injury. Because this mechanism is most strongly established in CAR-T cells and bispecific T-cell engagers, systemic cytokine release itself should not be assumed to represent a universal cross-therapy convergence node. Instead, downstream myeloid amplification and endothelial activation provide more plausible candidate interfaces through which systemic inflammation may converge with cardiovascular injury initiated by mechanistically distinct therapies.

### 4.3 Sterile danger sensing after primary cardiomyocyte or vascular injury

Sterile danger sensing provides a mechanistic bridge between primary cardiovascular tissue injury and secondary immune amplification. In cancer therapy-related cardiovascular toxicity, damage-associated molecular patterns (DAMPs) have been proposed as contributors to inflammatory cardiovascular injury after treatment-induced cellular stress or death (Klee et al., 2017). This mechanism is particularly relevant for therapies in which the initial insult is not primarily immune-mediated, such as cardiomyocyte stress, endothelial injury, mitochondrial dysfunction, DNA damage, oxidative stress, or payload-related tissue injury.

In injured cardiovascular tissues, DAMPs released from dying cells or damaged extracellular matrix can activate pattern-recognition receptor pathways, leading to cytokine and chemokine production and leukocyte recruitment (Frangogiannis, 2014b; de Haan et al., 2013). In the setting of cancer therapy, treatment-induced cell stress or death may similarly release DAMPs that activate inflammatory signaling within cardiovascular tissues (Klee et al., 2017). In this framework, sterile inflammation does not replace therapy-specific mechanisms of toxicity; rather, it explains how direct cellular injury can become coupled to a broader cardio-immune response.

The NLRP3 inflammasome is one of the best-studied sterile inflammatory sensors in cardio-oncology. A recent review summarized evidence linking mitochondrial dysfunction, oxidative stress, lysosomal damage, impaired autophagy, cellular senescence, DNA damage, and sterile inflammation to NLRP3 inflammasome activation in cancer therapy-associated cardiovascular injury (Mauro et al., 2023). However, NLRP3 signaling should not be described as uniformly harmful in every model. In one doxorubicin model, NLRP3 deficiency reduced macrophage IL-10 production and increased susceptibility to cardiotoxicity, whereas another juvenile mouse study found that resveratrol reduced cardiac NLRP3 inflammasome activation, systemic inflammation, and doxorubicin-induced cardiac injury (Kobayashi et al., 2016; Maayah et al., 2021). These apparently divergent findings suggest that inflammasome signaling may be shaped by cell type, developmental context, timing, and the balance between inflammatory injury and reparative immune regulation.

The cGAS–STING pathway represents another danger-sensing axis with relevance to therapy-induced cardiovascular injury. In a doxorubicin-induced cardiotoxicity model, doxorubicin activated cardiac endothelial cGAS–STING–IRF3 signaling, induced CD38 expression, and contributed to NAD depletion and mitochondrial dysfunction (Luo et al., 2023). Separately, a study of radiation-exposed human cardiac endothelial cells identified increased expression of cGAS–STING-associated inflammatory proteins, suggesting that this pathway may also contribute to radiation-induced endothelial inflammatory responses (Philipp et al., 2020). These data are especially relevant to the cardio-immune microenvironment because they place endothelial cells, rather than cardiomyocytes alone, at the interface between genotoxic stress and innate immune activation.

Therefore, sterile danger sensing should be interpreted as an injury-amplification module rather than a therapy-universal initiating mechanism. Its strongest mechanistic support currently comes from preclinical models, endothelial radiation-response studies, and broader cardio-oncology inflammation literature. For newer anticancer therapies such as ADCs, targeted agents, and combination regimens, the key question is not whether every drug activates the same DAMP, inflammasome, or cytosolic DNA-sensing pathway. Instead, the key question is whether mechanistically distinct therapies engage reproducible danger-sensing states that contribute to injury amplification rather than merely reflecting generic tissue damage and repair. The divergent NLRP3 findings emphasize that pathway activation alone is insufficient to establish a uniformly pathogenic convergence mechanism.

### 4.4 Myeloid and stromal reprogramming

Myeloid and stromal reprogramming represents a key mechanism through which acute inflammatory signals are converted into tissue-level cardiovascular remodeling. After myocardial injury, cardiac macrophage populations are reshaped by both local proliferation of resident macrophages and recruitment of circulating monocytes, indicating that the cardiac myeloid compartment is dynamic rather than fixed (Epelman et al., 2014; Dick et al., 2019). This distinction is important because resident and recruited macrophages may have different roles in inflammation, repair, and adverse remodeling.

Experimental myocardial injury studies have shown that infiltrating CCR2+ monocyte-derived macrophages can express inflammatory chemokines, cytokines, and genes associated with adverse cardiac remodeling (Bajpai et al., 2019). In contrast, self-renewing resident cardiac macrophages have been shown to limit adverse ventricular remodeling after myocardial infarction, supporting the concept that macrophage origin and activation state can influence the balance between repair and injury (Dick et al.). These findings provide a useful framework for cancer therapy-related cardiovascular toxicity, in which treatment-induced stress may alter the composition and function of local immune cells within cardiac tissue.

Direct human tissue evidence for myeloid and stromal involvement is strong in immune checkpoint inhibitor-associated myocarditis. Single-cell and spatial profiling of human checkpoint myocarditis showed increased frequencies and co-localization of cytotoxic T cells, conventional dendritic cells, and inflammatory fibroblasts in affected cardiac tissue (Blum et al., 2024). This supports a multicellular model in which T-cell injury is coupled to antigen presentation, stromal inflammation, and fibroblast-associated remodeling rather than acting as an isolated lymphocytic process.

Myeloid reprogramming is also relevant to T-cell–redirecting therapies. In experimental CAR-T models, recipient macrophages and monocytes have been identified as major sources of IL-1 and IL-6 during CRS (Giavridis et al., 2018; Norelli et al., 2018). This suggests that therapeutic T-cell activation can be amplified by innate immune cells, creating an inflammatory circuit that may secondarily affect endothelial cells, cardiomyocytes, and vascular tone.

Clonal hematopoiesis of indeterminate potential (CHIP) provides a further example of how altered hematopoietic cell states may influence cardiovascular injury. CHIP has been associated with increased cardiovascular risk, with experimental evidence implicating mutant hematopoietic cells and their inflammatory responses in cardiovascular disease (Jaiswal et al., 2017). In cancer therapy-related settings, experimental studies have shown that TP53-mutant clonal hematopoiesis can exacerbate doxorubicin-induced cardiotoxicity through enhanced neutrophil-mediated injury, while PPM1D-mutant clonal hematopoiesis can promote inflammation and adverse cardiac remodeling (Sano et al., 2021; Yura et al., 2021). These findings suggest that pre-existing or therapy-selected hematopoietic clones may modify cardiovascular susceptibility through altered inflammatory responses. However, whether comparable CHIP-associated myeloid states contribute to cardiovascular toxicity across mechanistically distinct novel anticancer therapies remains to be established.

Stromal cells provide the second arm of this reprogramming module. Single-cell profiling after myocardial injury has revealed dynamic changes across stromal, vascular, and immune cell populations, including fibroblast activation and inflammatory remodeling (Farbehi et al., 2019). Cardiac fibroblasts and extracellular matrix are not passive structural elements; they organize intercellular communication among cardiomyocytes, macrophages, endothelial cells, and other myocardial cell types (Bowers et al., 2022). In disease models, cardiac macrophage expansion and phenotypic changes can promote fibroblast activation, collagen deposition, myocardial stiffness, and diastolic dysfunction (Hulsmans et al., 2018).

In the context of novel anticancer therapies, myeloid and stromal reprogramming should therefore be viewed as a response module that links immune activation or primary cellular injury to longer-term tissue consequences. Its contribution is likely most evident in ICI myocarditis and CRS-associated T-cell therapies, but it may also shape recovery after targeted therapy-, ADC-, or radiotherapy-related cardiomyocyte and endothelial injury. The key unresolved issue is whether these therapies produce distinct myeloid–stromal signatures in human cardiovascular tissue or whether they converge on a limited set of generic inflammatory repair programs. Until reproducible cross-therapy states and their temporal relationship to cardiovascular injury are demonstrated, myeloid–stromal reprogramming should therefore be regarded as a candidate convergence module rather than an established shared pathogenic mechanism.

### 4.5 Endothelial–immune coupling and chronic cardiovascular remodeling

Endothelial–immune coupling provides a vascular route through which systemic inflammation and therapy-induced tissue stress are translated into cardiovascular injury. The vascular endothelium regulates vascular tone, permeability, leukocyte trafficking, coagulation, and microvascular perfusion (Terwoord et al., 2022). Anti-cancer therapy-related vascular toxicity is frequently mediated by endothelial injury and can manifest as hypertension, vasospastic or thrombotic ischemic events, venous thromboembolism, capillary leak, and microvascular dysfunction (Herrmann et al., 2016; Campia, 2020). This mechanism is important because cardiovascular toxicity induced by novel anticancer therapies is not limited to cardiomyocyte dysfunction or myocarditis.

Immune checkpoint inhibition provides one example of how immune activation may affect the vascular compartment. In a matched cohort study, exposure to ICIs was associated with a higher risk of atherosclerotic cardiovascular events and greater progression of total aortic plaque volume (Drobni et al., 2020). Experimental and translational data further suggest that checkpoint inhibition can aggravate T-cell-driven plaque inflammation and promote an inflammatory plaque phenotype (Poels et al., 2020). These findings indicate that loss of immune restraint may affect not only myocardial immune tolerance but also vascular inflammation and atherosclerotic disease activity.

T-cell–redirecting therapies illustrate a different route into endothelial–immune coupling. Severe CRS after CD19 CAR-T-cell therapy has been associated with hemodynamic instability, capillary leak, consumptive coagulopathy, and increased endothelial activation markers such as angiopoietin-2 and von Willebrand factor (Hay et al., 2017). In this context, endothelial activation may help convert circulating cytokine release into vascular permeability, impaired tissue perfusion, coagulation activation, and secondary myocardial stress.

Radiotherapy provides a further example in which endothelial injury can evolve into chronic cardiovascular remodeling. Contemporary reviews and cardio-oncology consensus statements identify endothelial cell damage, oxidative stress, inflammatory and profibrotic cytokine release, accelerated atherosclerosis, pro-thrombotic changes, and fibrosis of myocardial, pericardial, valvular, and conduction tissues as key mechanisms of radiation-induced cardiovascular disease (Koutroumpak et al., 2022; Mitchell et al., 2021). These mechanisms are particularly relevant to combination regimens, in which radiation-induced local tissue injury may coexist with systemic immune activation or targeted therapy-related vascular stress.

The chronic consequences of endothelial–immune coupling are not restricted to vascular events. Persistent inflammation, microvascular dysfunction, and fibroblast activation can promote extracellular-matrix deposition and myocardial fibrosis. Diffuse myocardial fibrosis contributes to adverse cardiac function and clinical outcomes in heart failure, whereas fibrotic remodeling can create an arrhythmogenic substrate by disrupting electrical connectivity and tissue conduction (López et al., 2021; de Jong et al., 2011). Inflammatory cells may also contribute to rhythm disturbances by altering tissue composition, producing cytokines, promoting fibrosis, or interacting with cardiomyocytes (Grune et al., 2021).

Thus, endothelial–immune coupling links acute cardio-immune injury to chronic cardiovascular phenotypes. Across modern anticancer therapies, this module may help explain why initially distinct insults can ultimately manifest as hypertension, vascular inflammation, thrombosis, myocardial fibrosis, arrhythmia, and heart failure. Its relative contribution is likely greatest in angiogenesis inhibition, CAR-T-associated CRS, immune checkpoint inhibitor-associated vascular inflammation, radiotherapy, and combination regimens. However, although clinical and translational evidence supports endothelial perturbation across several treatment contexts, direct human cardiovascular tissue evidence remains limited, and cross-therapy causal perturbation of a common endothelial–immune node has not been established. Endothelial–immune coupling should therefore be considered a plausible candidate convergence module whose predictive and causal relevance requires prospective validation.

## 5 Translational implications: from cardio-immune mechanisms to precision cardio-oncology

The cardio-immune framework has practical implications for risk assessment, monitoring, and intervention in patients receiving novel anticancer therapies. Because different treatment classes are associated with distinct cardiovascular toxicity profiles, translational strategies should be aligned with treatment exposure and dominant injury context rather than based on a uniform surveillance model (Lyon et al., 2022; Raisi-Estabragh et al., 2024). Standard cardiac tools, including biomarkers, electrocardiography, echocardiography, and cardiac magnetic resonance, remain essential, but their interpretation should be adapted to the dominant toxicity context (Lyon et al., 2022; Alvarez-Cardona et al., 2020; Čelutkienė et al., 2020). For example, elevations in cardiac troponins or natriuretic peptides during cancer therapy should be interpreted in relation to treatment exposure, clinical presentation, electrocardiographic findings, and cardiac imaging, rather than as a treatment-independent marker of a single pathogenic mechanism (Lyon et al., 2022; Alvarez-Cardona et al., 2020). The cardio-immune framework is not intended to replace guideline-based care, but to distinguish proximal injury from downstream amplification or nonspecific repair. Its potential clinical applications remain to be prospectively validated.

### 5.1 Cardio-immune biomarkers and risk stratification

Cardio-immune risk stratification should be therapy-specific rather than based on a uniform surveillance algorithm. The HFA–ICOS position statement proposed baseline cardiovascular risk stratification tools for several cardiotoxic cancer therapy classes and emphasized that clinical history and baseline investigations should be tailored to the planned cancer treatment (Lyon et al., 2020). Within this framework, conventional cardiac biomarkers remain foundational but nonspecific: cardiac troponins are markers of myocardial injury, whereas natriuretic peptides reflect myocardial stress or hemodynamic congestion (Alvarez-Cardona et al., 2020). Accordingly, these biomarkers can identify cardiovascular injury or stress but cannot by themselves establish whether immune activation initiated the injury, amplified another primary insult, or emerged secondarily during tissue repair. In this context, conventional cardiac biomarkers and established imaging modalities remain the basis of clinical assessment, whereas emerging endothelial, immune, and omics-derived biomarkers remain investigational and require prospective validation.

For immune checkpoint inhibitor therapy, biomarker-based risk stratification should be integrated with clinical and imaging evidence of myocarditis. ICI-associated myocarditis is an uncommon but high-risk toxicity, and troponin elevation is frequently used as part of the diagnostic and monitoring evaluation (Lyon et al., 2022; Mahmood et al., 2018). However, cardiac function may be preserved despite clinically important disease. In patients with ICI-related myocarditis, global longitudinal strain was associated with major adverse cardiac events even among patients with preserved left ventricular ejection fraction, supporting the value of imaging-based risk refinement beyond conventional systolic function (Awadalla et al., 2020).

For CAR-T-cell therapy and other T-cell–redirecting therapies, cardiovascular biomarkers should be interpreted within the context of CRS and systemic inflammatory toxicity. In adults treated with CAR-T cells, CRS, troponin elevation, and cardiovascular events showed a graded relationship, and severe CRS has been associated with subsequent major adverse cardiovascular events (Alvi et al., 2019; Lefebvre et al., 2020). Within the convergence framework, an important unresolved question is whether markers of myeloid or endothelial activation provide information about cardiovascular risk beyond CRS severity and conventional measures of myocardial injury. For example, prospective studies could test whether endothelial activation markers such as angiopoietin-2 or von Willebrand factor identify patients in whom systemic inflammation is being translated into cardiovascular injury rather than simply reflecting severe CRS(56).

For vascular-toxic therapies, especially VEGF or angiogenesis pathway inhibition, risk stratification should prioritize vascular phenotype in addition to conventional cardiac biomarkers. Cancer therapy-related vascular toxicity includes arterial events, venous thromboembolism, pulmonary hypertension, endothelial injury, and other vascular complications (Her and rmann, 2020).

For antibody–drug conjugates, biomarker interpretation should remain phenotype-driven and therapy-specific. ADC-related toxicity may be influenced by target-independent uptake, linker instability, premature payload release, payload class, and tissue exposure rather than a uniform immune-mediated mechanism (Mahalingaiah et al., 2019; Donaghy, 2016). Clinical evaluation should therefore account for prior cardiotoxic therapy exposure, baseline cardiovascular vulnerability, and the observed cardiovascular phenotype.

Overall, cardio-immune risk stratification should integrate baseline cardiovascular vulnerability, treatment class, conventional cardiac biomarkers, inflammatory toxicity grade, vascular phenotype, and imaging-based cardiac assessment. Omics-based biomarker discovery may help identify molecularly targeted predictors of cancer therapy-related cardiovascular toxicity, but such approaches remain investigational and require clinical validation before routine use (Rhee et al., 2020).

### 5.2 Mechanism-guided monitoring and intervention

Mechanism-guided monitoring and intervention should match the dominant injury context of each treatment rather than apply a uniform cardioprotective strategy across all novel anticancer therapies. Current guideline-directed management remains the clinical standard: suspected ICI myocarditis requires rapid diagnostic evaluation and immunosuppressive treatment when indicated; CAR-T-associated cardiovascular toxicity should be assessed together with CRS severity and hemodynamic status; VEGF-pathway inhibition requires active blood-pressure and vascular-risk management; and HER2-directed therapy requires surveillance for ventricular dysfunction (Lyon et al., 2022; Schneider et al., 2021; Lee et al., 2019; Maitland et al., 2010). The cardio-immune framework does not alter these established recommendations at present.

For ICI-associated myocarditis, mechanism-guided immunomodulation is also being evaluated clinically. The ongoing phase 3 ATRIUM trial (NCT05335928) is a randomized, double-blind, placebo-controlled study evaluating abatacept added to corticosteroid therapy in hospitalized patients with ICI-associated myocarditis, with major adverse cardiac events as the primary endpoint; results have not yet been reported (Reynolds et al., 2023). In addition, Salem and colleagues reported a treatment-intensification strategy incorporating abatacept and ruxolitinib in severe ICI-associated myocarditis (Salem et al., 2023). These approaches provide clinically relevant examples of therapeutic strategies directed at checkpoint-related T-cell activation and downstream inflammatory signaling, although their role in routine management requires further prospective validation.

Its potential added value is to identify circumstances in which monitoring could eventually be escalated according to the biological route through which toxicity is evolving. For example, an inflammatory myocardial phenotype during ICI therapy may justify attention to immune activation and tissue injury, whereas cardiovascular abnormalities during severe CRS may require simultaneous assessment of myocardial injury, hemodynamic stress, and endothelial dysfunction. Conversely, ventricular dysfunction during HER2-directed therapy should not be presumed to require immune-directed intervention simply because secondary inflammatory responses are detectable.

A more stringent translational test of the framework is whether a candidate convergence node provides actionable information beyond existing clinical variables. For CAR-T therapy, for example, prospective studies should determine whether endothelial or myeloid activation improves prediction of cardiovascular events beyond CRS grade, troponin, vital signs, and ventricular function. Similarly, for therapies producing primary cardiomyocyte or vascular injury, longitudinal profiling should determine whether emergence of a defined inflammatory response identifies patients progressing from reversible cellular stress to persistent injury or remodeling.

At present, these applications should be considered evidence gaps rather than recommendations for routine care. A candidate convergence node would become clinically relevant only if its measurement improves risk classification or treatment selection beyond conventional assessment and, ultimately, if modulation of that node improves cardiovascular outcomes without compromising anticancer efficacy. Framework-derived candidate convergence nodes and their testable predictions are summarized in Table 2.

**TABLE 2:** Framework-derived testable predictions for cardio-immune convergence.

Mechanistic domain	Candidate convergence nodes	Therapy contexts	Testable prediction	References
Immune tolerance breakdown	Antigen-specific autoreactive T-cell responses	Predominantly ICI myocarditis; cross-therapy convergence not established	Identification of a comparable antigen-specific cardio-immune response in another mechanistically distinct therapy would support convergence; restriction to ICI would indicate a therapy-specific initiating mechanism. In ICI myocarditis, mechanism-directed immunomodulation is being investigated clinically, although this does not establish cross-therapy convergence	Wei et al. (2021), Lv et al. (2011), Axelrod et al. (2022), Won et al. (2022), Blum et al. (2024), Reynolds et al. (2023), Salem et al. (2023)
Cytokine-driven systemic inflammation	IL-1/IL-6-centered inflammatory signaling	CAR-T and other highly inflammatory treatment contexts; cross-therapy cardiovascular convergence remains uncertain	A shared cytokine-linked cardiovascular response should predict or modify cardiac injury across ≥2 mechanistically distinct therapy classes, beyond overall systemic inflammatory severity	Morris et al. (2022), Giavridis et al. (2018), Norelli et al. (2018), Maleki et al. (2024), Hay et al. (2017)
Sterile danger sensing	cGAS–STING; context-dependent NLRP3 signaling	Doxorubicin; radiotherapy	A true shared node should be activated before injury amplification, and its perturbation should modify cardiovascular toxicity across ≥2 treatment contexts; late activation alone would favor a generic injury response	Mauro et al. (2023), Kobayashi et al. (2016), Maayah et al. (2021), Luo et al. (2023), Philipp et al. (2020)
Myeloid and stromal reprogramming	Monocyte/macrophage and macrophage–fibroblast response states	ICI; CAR-T; primary myocardial injury contexts	Reproducible cellular states should occur across distinct therapies and influence injury persistence or remodeling; changes confined to late repair would favor nonspecific tissue repair	Blum et al. (2024), Giavridis et al. (2018), Norelli et al. (2018), Dick et al. (2019), Bajpai et al. (2019), Jaiswal et al. (2017), Sano et al. (2021), Yura et al. (2021), Farbehi et al. (2019), Bowers et al. (2022), Hulsmans et al. (2018)
Endothelial–immune coupling	Endothelial activation and endothelial–leukocyte signaling	CAR-T; VEGF/VEGFR inhibition; ICI; radiotherapy	A shared endothelial response should predict toxicity beyond therapy-specific severity measures, and perturbation of a common node should alter cardiovascular injury across ≥2 treatment contexts	Hay et al. (2017), Philipp et al. (2020), Terwoord et al. (2022), Campia (2020), Drobni et al. (2020), Poels et al. (2020), Mitchell et al. (2021)

Candidate convergence nodes are not necessarily established cross-therapy mechanisms. Operationally, true convergence requires engagement of a shared cellular or molecular node downstream of mechanistically distinct proximal insults, a temporal relationship with the cardiovascular injury trajectory, and ideally modification of toxicity by perturbing that node across at least two treatment contexts. Responses appearing only after established tissue injury without such temporal or causal relevance are more appropriately interpreted as generic injury–repair responses. Abbreviations: ICI, immune checkpoint inhibitor; VEGF, vascular endothelial growth factor; VEGFR, vascular endothelial growth factor receptor.

### 5.3 Multi-omics and precision cardio-oncology

Precision cardio-oncology will require a shift from isolated biomarkers toward longitudinal, mechanism-informed integration of clinical, imaging, and molecular data. Omics-based approaches, including genomics, transcriptomics, proteomics, metabolomics, and radiomics, may help identify molecular signatures for early detection, risk stratification, and individualized monitoring of cancer therapy-related cardiovascular toxicity (Kuang et al., 2024). Because cardiovascular toxicity across modern anticancer therapies is heterogeneous, no single biomarker or omics layer is likely to provide sufficient diagnostic or prognostic information. Candidate omics signatures should therefore undergo reproducibility assessment, validation in independent cohorts, and evaluation of their incremental predictive value and clinical utility before routine clinical adoption (Rhee et al., 2020; Kuang et al., 2024; Hlatky et al., 2009).

For cardio-immune toxicity, single-cell and deep immunophenotyping approaches may be particularly informative. Techniques such as single-cell RNA sequencing (scRNA-seq), time-of-flight mass cytometry (CyTOF), single-cell T-cell receptor sequencing (scTCR-seq), and cellular indexing of transcriptomes and epitopes by sequencing (CITE-seq) can characterize immune-cell states, transcriptomic programs, surface protein expression, and antigen-receptor repertoires in immune therapy-related cardiotoxicity (Huang et al., 2022). Candidate features for future biomarker development include cytotoxic T-cell and conventional dendritic-cell populations, as well as inflammatory fibroblast states identified in human ICI-associated myocarditis (Blum et al., 2024), and endothelial activation markers such as angiopoietin-2 and von Willebrand factor associated with severe CAR-T-related CRS(56). These features remain investigational, and their potential value lies in whether their temporal and treatment-specific patterns can distinguish immune initiation or injury amplification from nonspecific responses to established tissue injury. Rather than applying these technologies as exploratory profiling tools alone, the convergence framework provides specific biological questions that they can test.

First, paired T-cell receptor profiling across tumor, blood, and myocardium could determine whether expanded or shared clonotypes support antigen-directed immune initiation, as suggested in ICI-associated myocarditis, and how these clonotypes relate temporally to downstream myeloid and stromal responses (Johnson et al., 2016; Blum et al., 2024). Second, longitudinal single-cell or proteomic profiling across mechanistically distinct treatment classes could test whether reproducible myeloid or endothelial states emerge before cardiovascular toxicity progresses, thereby distinguishing candidate convergence modules from late nonspecific repair responses. Third, serial molecular profiling could determine whether activation of these candidate modules predicts persistence, resolution, or remodeling beyond conventional markers such as troponin, natriuretic peptides, CRS grade, and imaging abnormalities.

These questions also provide a direct way to test the operational definition proposed in Section 2. Evidence for convergence would be strengthened if mechanistically distinct therapies retained treatment-specific proximal signatures while developing reproducible shared downstream cellular states, particularly when those states preceded toxicity progression. Conversely, molecular programs that appear only after established tissue injury or that simply reproduce generic wound-healing responses would provide weaker evidence for mechanistically meaningful convergence.

Future cardio-oncology risk models may incorporate omics-derived variables, but they should be developed and reported according to rigorous prediction-model standards. Model performance should be assessed using discrimination, calibration, external validation, and clinical utility, and prediction tools using machine learning or artificial intelligence should follow updated reporting guidance for regression- or AI-based clinical prediction models (Collins et al., 2015; Collins et al., 2024). If genomic predictors are included, performance should also be evaluated across ancestry groups because polygenic risk prediction can vary substantially across populations (Martin et al., 2019).

Overall, multi-omics should be used to test mechanism-based hypotheses rather than simply expand the number of candidate biomarkers. Its most immediate value within the cardio-immune framework is to distinguish immune initiation from downstream amplification, identify reproducible cross-therapy response states, and determine whether those states add predictive or therapeutic information beyond established clinical assessment.

## 6 Challenges and future perspectives

Despite growing interest in cardio-immune mechanisms, several challenges limit the current understanding of cardiovascular toxicity induced by novel anticancer therapies. First, the evidence base remains uneven across treatment classes. Immune checkpoint inhibitor-associated myocarditis is supported by human pathological, immunological, and spatial profiling studies that demonstrate myocardial immune-cell infiltration, antigen-directed T-cell responses, and multicellular inflammatory niches within affected cardiac tissue (Johnson et al., 2016; Axelrod et al., 2022; Blum et al., 2024). By contrast, cardio-immune mechanisms in targeted therapies, ADCs, angiogenesis inhibitors, and selected combination regimens are less directly defined in human cardiac tissue studies and are more often discussed on the basis of pathway biology, preclinical models, clinical toxicity patterns, pharmacovigilance analyses, or biomarker-based evidence (Touyz and Herrmann, 2018; Force et al., 2007; Mahalingaiah et al., 2019; Inno et al., 2024). This imbalance should be acknowledged to avoid overstating a unified immune mechanism across all novel anticancer therapies.

Second, human cardiovascular tissue evidence remains scarce. Most mechanistic studies rely on peripheral blood, animal models, cell culture systems, or retrospective clinical datasets. Although these approaches are valuable, they may not fully capture the local cardio-immune microenvironment, where immune cells, cardiomyocytes, endothelial cells, fibroblasts, extracellular matrix, and vascular structures interact dynamically. Future studies should prioritize longitudinal human datasets that integrate blood-based immune profiling with cardiac imaging, clinical phenotyping, and, when available, tissue-level analysis. Human induced pluripotent stem cell-derived cardiomyocytes (iPSC-CMs), engineered heart tissues, and co-culture systems incorporating immune or endothelial cells provide complementary experimental platforms for investigating these mechanisms. Human iPSC-CMs have been used to model cardiomyocyte dysfunction induced by anticancer therapies, including trastuzumab, while engineered heart tissues can capture aspects of contractile dysfunction and tissue-level responses to cardiotoxic injury (Kitani et al., 2019; Linders et al., 2023). Moreover, co-culture models combining human iPSC-CMs with immune cells have been developed to investigate ICI-associated myocarditis and immune-mediated cardiomyocyte injury (Jensen et al., 2024). By comparing cardiomyocyte-only cultures with immune- or endothelial-cell co-cultures, these platforms could help distinguish direct cardiomyocyte injury, endothelial-mediated injury, and secondary immune amplification under controlled experimental conditions. Importantly, controlled genetic or pharmacological perturbation of candidate signaling nodes across mechanistically distinct treatment exposures could provide functional evidence of whether the same downstream response module causally modifies cardiovascular injury across different treatment contexts. Nevertheless, the limited maturation and cellular complexity of current *in vitro* models should be considered when translating these findings to human cardiovascular toxicity.

Third, it remains difficult to distinguish immune mechanisms that drive cardiovascular injury from those that simply accompany tissue damage. For example, macrophage activation, cytokine release, endothelial dysfunction, and fibroblast activation may be pathogenic in some contexts but reparative or compensatory in others. The same pathway may also have different effects depending on timing, cell type, treatment exposure, and baseline cardiovascular vulnerability. Important boundary conditions include trastuzumab-associated cardiac dysfunction, in which cardiomyocyte HER2/ErbB2-dependent mechanisms are well established (Grazette et al., 2004; Crone et al., 2002), whereas direct human histopathological evidence for prominent inflammatory myocardial infiltration is limited (Ewer et al., 2005; Slavcheva and Angelov, 2023), arguing against its interpretation as an established immune-initiated toxicity. NLRP3 signaling provides a different caution, because its effects in doxorubicin models are context dependent, with both protective and detrimental roles reported (Kobayashi et al., 2016; Maayah et al., 2021). Combination effects are likewise heterogeneous: ICI plus chemotherapy has been associated with increased cardiotoxicity (Liu et al., 2022), whereas ICI plus angiogenesis inhibitors increased severe hypertension without significantly increasing acute vascular events (Inno et al., 2024), cautioning against assuming uniform synergistic cardiotoxicity. Together, these observations indicate that the convergence framework is most applicable when a shared downstream response can be temporally and mechanistically linked to the injury trajectory; when such evidence is absent, overlapping inflammation or remodeling may instead represent nonspecific tissue repair. Future research should therefore move beyond static measurement of inflammatory markers and focus on temporal immune trajectories, cell-specific functions, and causal validation.

Fourth, a major translational challenge is how to suppress maladaptive cardiovascular inflammation without compromising antitumor efficacy. This issue is particularly important for ICIs, CAR-T cells, and bispecific T-cell engagers, where immune activation is central to therapeutic benefit. Broad immunosuppression may reduce cardiovascular injury but could also interfere with cancer control. More selective approaches will require better identification of the immune pathways that are dispensable for tumor response but critical for cardiovascular toxicity.

Finally, precision cardio-oncology will require therapy-specific rather than one-size-fits-all models. ICI myocarditis, CAR-T-related cardiovascular events, VEGF inhibitor-associated vascular toxicity, ADC-related tissue injury, and radiation-induced cardiovascular disease may share downstream inflammatory and remodeling features, but their proximal triggers differ. Future studies should determine which candidate convergence modules are reproducible across mechanistically distinct therapies and whether their modulation alters cardiovascular injury. Overall, the key challenge is not to prove that all anticancer therapies cause cardiovascular toxicity through the same immune pathway. Rather, the field should determine when therapy-specific injury engages meaningful shared cardio-immune responses and when apparent overlap reflects generic tissue repair.
